# Improving Diagnosis and Prognosis in Acute Severe Brain Injury: A Multimodal Imaging Protocol

**DOI:** 10.3389/fneur.2021.757219

**Published:** 2021-12-06

**Authors:** Karnig Kazazian, Loretta Norton, Geoffrey Laforge, Androu Abdalmalak, Teneille E. Gofton, Derek Debicki, Marat Slessarev, Sarah Hollywood, Keith St. Lawrence, Adrian M. Owen

**Affiliations:** ^1^Graduate Program in Neuroscience, Schulich School of Medicine and Dentistry, Western University, London, ON, Canada; ^2^Brain and Mind Institute, Western University, London, ON, Canada; ^3^Department of Psychology, King's University College at Western University, London, ON, Canada; ^4^Department of Psychology, Western University, London, ON, Canada; ^5^Department of Physiology and Pharmacology, Schulich School of Medicine and Dentistry, Western University, London, ON, Canada; ^6^Department of Clinical Neurological Sciences, Schulich School of Medicine and Dentistry, Western University, London, ON, Canada; ^7^Department of Medicine, Schulich School of Medicine and Dentistry, Western University, London, ON, Canada; ^8^Department of Medical Biophysics, Schulich School of Medicine and Dentistry, Western University, London, ON, Canada

**Keywords:** coma, intensive care unit, functional near infrared spectroscopy (fNIRS), functional magnetic resonance imaging (fMRI), electroencephalography

## Abstract

Multi-modal neuroimaging techniques have the potential to dramatically improve the diagnosis of the level consciousness and prognostication of neurological outcome for patients with severe brain injury in the intensive care unit (ICU). This protocol describes a study that will utilize functional Magnetic Resonance Imaging (fMRI), electroencephalography (EEG), and functional Near Infrared Spectroscopy (fNIRS) to measure and map the brain activity of acute critically ill patients. Our goal is to investigate whether these modalities can provide objective and quantifiable indicators of good neurological outcome and reliably detect conscious awareness. To this end, we will conduct a prospective longitudinal cohort study to validate the prognostic and diagnostic utility of neuroimaging techniques in the ICU. We will recruit 350 individuals from two ICUs over the course of 7 years. Participants will undergo fMRI, EEG, and fNIRS testing several times over the first 10 days of care to assess for residual cognitive function and evidence of covert awareness. Patients who regain behavioral awareness will be asked to complete web-based neurocognitive tests for 1 year, as well as return for follow up neuroimaging to determine which acute imaging features are most predictive of cognitive and functional recovery. Ultimately, multi-modal neuroimaging techniques may improve the clinical assessments of patients' level of consciousness, aid in the prediction of outcome, and facilitate efforts to find interventional methods that improve recovery and quality of life.

## Introduction

Severe acute brain injury is a medical emergency that requires immediate admission to an intensive care unit (ICU) for life-sustaining therapies. Little is known about residual cognitive function in the first few days following injury, which makes both the accurate diagnosis of the level of consciousness and the prognostication of neurological outcome very challenging. Current prognostic tools focus on predicting a poor neurological outcome (a clinical outcome no better than vegetative state or severe disability with total dependency) (1). As it stands, there are no accepted clinical measures used in standard practice that can determine the likelihood of good functional recovery (recovery that allows for sufficient function for independent activities of daily life) (2). However, recent research has suggested that advanced neuroimaging methods, such as structural imaging (diffusion-weighted and diffusion-tensor) may provide additional prognostic information that is not currently attainable using standard clinical measures (3–5). There is also a lack of reliable diagnostic tools for assessing the level of consciousness a patient retains while in the ICU. Subjective bedside behavioral assessments are primarily used, which can be confounded by sedative and analgesic medication that are given to patients to tolerate mechanical ventilation, facial and ocular injuries, and their inter-rater reliability has been called into question (6, 7). Accurately classifying the level of consciousness at the bedside is imperative, as a growing body of research suggests that these patients may have a period of dissociation between regaining awareness and behavioral responsiveness (8, 9). To this end, there is a need to develop methods that can be deployed in the ICU to accurately assess residual cognitive function in the early days of injury, thereby improving patient care and inform clinical decision making.

Multi-modal imaging techniques may offer new ways for advancing the prognostication and diagnosis of critically ill patients in the ICU. Functional magnetic resonance imaging (fMRI) has been used to assess the integrity of passive perceptual and higher-order cognitive abilities in chronic disorders of consciousness, such as patients in a vegetative state and unresponsive wakefulness syndrome, who regain wakefulness in the months to years following acute injury but lack overt behavioral function (10, 11). Further, fMRI studies have shown that 15–20% of chronic disorders of consciousness patients can willfully modulate their brain activity in response to command following tasks; suggesting a level of covert awareness that may not be apparent clinically (12, 13). Importantly, fMRI findings have demonstrated prognostic value, as activity in higher-order integrative cortical areas to external stimuli has been associated with improvements in functional recovery (14). While a substantial amount of fMRI research has examined brain activity in chronic patients with disorders of consciousness, few studies have explored its use in an acute intensive care setting (9, 15–18). As such, the full prognostic and diagnostic efficacy of fMRI in this patient population has yet to be determined.

Portable and low-risk modalities, such as electroencephalography (EEG) and functional near infrared spectroscopy (fNIRS), are promising neuroimaging techniques that can be used to assess cortical function at a patient's bedside. High-density EEG is low-cost and readily available in most clinical settings. In this way, EEG is ideal for routine and repetitive assessments of neural function in patients with severe brain injury. Moreover, EEG has been used extensively in patients with chronic disorders of consciousness to assess residual cognitive function (19, 20). In an acute setting, neural responses have shown some promise in predicting outcomes by assessing basic auditory function and covert speech comprehension (21, 22). FNIRS is also an attractive option for assessing brain activity in the ICU and is often considered the optical equivalent of fMRI. This method is a non-invasive optical technique that maps local changes in neural blood oxygenation in response to stimuli and at rest. Near infrared spectroscopy is a feasible tool for detecting neural activity following acute brain injury (23) and has been used as a communication modality with non-responsive patients in the ICU (24).

Utilizing a variety of imaging techniques (fMRI, EEG, fNIRS) together to assess neurophysiologic function offers new opportunities to study the brain following severe injury. Indeed, multiple neuroimaging modalities and tasks increase the chances of detecting residual cognitive function in patients with chronic disorders of consciousness (25). In an acute setting, one specific method may not be suitable for all patients, as the injuries of some individuals will render them ineligible for testing with certain neuroimaging modalities. For example, patients with metallic implants and raised intracranial pressure will be incompatible with fMRI, craniotomies and drains can impede EEG and fNIRS placement leading to abnormal recordings, and large bleeds can prevent the fNIRS probes from detecting a reliable signal.

This research protocol outlines a novel multimodal imaging program using all three imaging methods (fMRI, EEG, fNIRS) that aims to improve the diagnosis and prognosis of acutely unresponsive, brain injured patients. Critically ill patients will undergo testing several times over the course of the first 10 days of ICU stay to meet the following objectives:

1. To establish whether fMRI can aid prognostication in acute disorders of consciousness.
2. To establish whether fMRI measures of covert awareness can be used in the diagnosis of acute disorders of consciousness in the ICU.
3. To develop bedside methods (EEG, fNIRS) for assessing residual cognitive function and covert awareness in the ICU and establish whether these measures can supplement diagnosis and prognostication.
4. To Explore the use of bedside technology for communicating with select patients with acute brain injury.

The overarching hypothesis is that a subset of critically ill patients will show detectable neural responses to external stimuli and that this activity will be related to improved clinical outcomes and the recovery from acute brain injury. We also predict that a proportion of patients will be able to willfully modulate their brain activity in response to command following tasks, suggesting a level of covert awareness that may not be detectable with clinical examination alone. We anticipate that this multimodal research program will provide critical insight into neural function following severe brain injury and ultimately allow clinicians to render a more accurate diagnosis and prognosis for patients in the ICU.

## Materials and Methods

### Ethics

Ethical approval for this research study was obtained by the Health Sciences Research Ethics Board of Western University (project identification number: 114967). All study procedures will be performed per relevant guidelines and regulations and in accordance to the 1964 Declaration of Helsinki. Written informed consent will be obtained by the next of kin, as the patient population of this study will be unable to consent for acute imaging procedures. Patients will be asked to reconsent to the study if they regain the capacity to provide consent before longitudinal cognitive testing and imaging.

### Patient Selection, Recruitment, and Study Sites

This study will take place at London Health Sciences Centre, in London, Canada. Overall, 350 unresponsive brain injured patients between the ages of 18–75 will be identified in two ICUs over 7 years. We will screen and recruit from the Medical-Surgical ICU and the Critical Care Trauma Centre at University and Victoria Hospital in London, Canada. All patients with a primary brain injury will be screened for this study. Patients will be included if they have suffered a brain injury that has rendered them unresponsive (Glasgow Coma Scale < 8 in the absence of sedation). The clinicians involved in this study (TG, DD, MS) will confirm whether a patient is clinically considered to be unresponsive (does not obey verbal commands). This will be assessed via bedside examinations of consciousness such as the Glasgow Coma Scale. Eligible brain injuries include (but not limited to) traumatic brain injury, anoxic brain injury, stroke, subarachnoid hemorrhage; are acutely ill with required hospitalization in the ICU; and do have a pre-existing diagnosed neurological disorder that would impair cognition before ICU admission (i.e., Alzheimer's disease, Parkinson's disease, Huntington's disease). For MRI purposes, patients must be medically stable and deemed to be at low risk of deterioration during imaging procedures or transport to and from the MRI unit. Patients will be excluded if deemed medically unsuitable, have status epilepticus, or show evidence of overt command following and are responsive to external stimuli. For MRI testing, patients with unstable cardiac or respiratory status, intraorbital foreign bodies in or close to the retina, or any other contraindications will be excluded. Patients who do not meet in inclusion criteria for MRI will still be eligible for EEG and fNIRS testing. For EEG and fNIRS testing, patients with head injuries that would impede electrode and probe placement on the scalp will also be excluded. Specific to fNIRS testing, patients with hemorrhages will also be excluded if the bleed is large enough to affect the data quality which may result in a loss of signal for the probes located over the bleed. Finally, patients will be withdrawn from the study if GCS improves beyond eight prior to data collection.

#### Healthy Controls

Up to 50 age and sex matched healthy controls will be recruited in this study. Data acquired from these participants will used as a reference of typical neural activity for the neuroimaging paradigms in this study.

#### Feasibility

The sample size for this study was determined based on feasibility. We accounted for the estimated ICU survival rates for various common severe brain injuries, loss of data due to poor quality (~5–10%), and the potential of withdrawal from study procedures before, during, or after testing commences for various reasons (patient regains awareness before imaging procedures are complete, decline of reconsent, unexpected medical complications). Based on admission information obtained from support services at London Health Sciences Centre, we can expect approximately 550–650 patients admitted into the ICU with a life threating brain injury that requires mechanical ventilation each year. To this end, over 7 years we can expect approximately 4,200 patients that may be eligible for this study. As such, we are confident that 350 patients is an appropriate and feasible number for this study. Furthermore, this project will have a fulltime research coordinator who will work closely with the bedside nurses and attending physicians to schedule and ensure testing takes place at the correct and appropriate time. The research coordinator will also schedule and administer follow-up cognitive testing and telephone questionnaires.

### Protocol

#### Acute Imaging

**Figure 1:**
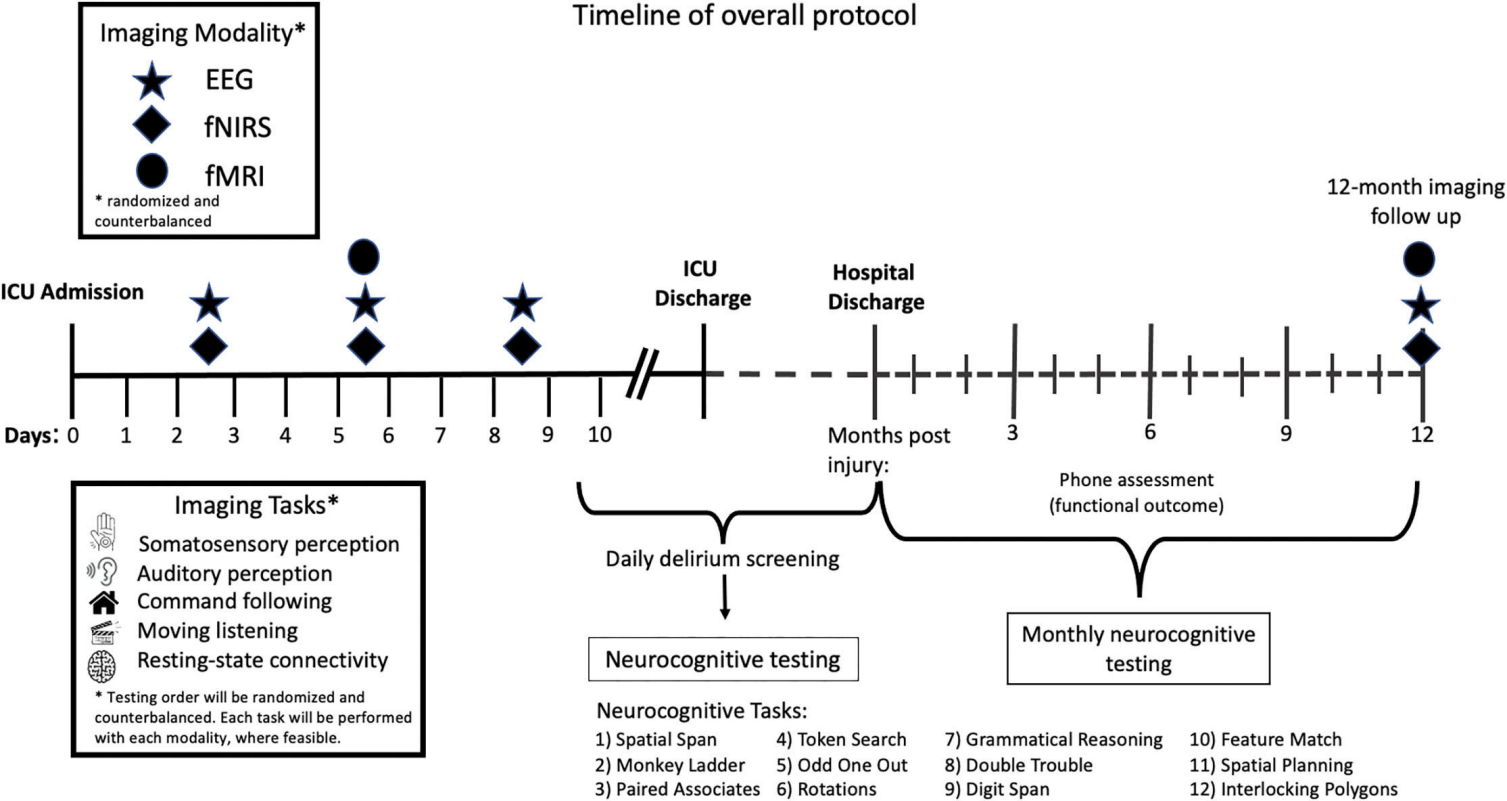
Timeline of overall protocol. After 2–3 days in the ICU, patients will undergo both EEG and fNIRS testing. Between days 4–6 participants will undergo EEG, fNIRS, and fMRI testing. EEG and fNIRS testing will be repeated between days 7 and 10. The order of stimuli presented will be randomized and counterbalanced across all imaging modalities and time points. The order in which imaging modalities will be used at each time point across the timeline will also be counterbalanced and randomized. Patients will undergo neurocognitive testing using the Cambridge Brain Sciences (CBS) cognitive battery once in hospital when delirium has resolved, and then monthly for up to 12 months post injury. Phone assessments to assess functional outcome will take place 3-, 6-, and 12-months post injury.

Study procedures will commence on days 2–3 after ICU admission with both EEG and fNIRS testing for a total test duration of 2 h within the 48-h period. These 2 h of testing will occur at some point on either day 2 or 3 of ICU admission. Between days 4–6, eligible participants will undergo fMRI, EEG, and fNIRS testing. Where possible, fMRI will occur immediately following clinically required structural imaging. The total test duration will be 3 h in the 72-h period, meaning that 3 h of testing will occur across day 5–7 of ICU stay. EEG and fNIRS testing will be repeated between days 7–10 with a testing duration of 2 h. The order of fNIRS and EEG testing will be randomized and counterbalanced for each time point. Stimuli will also be presented in a randomized fashion. Where clinically feasible, all stimuli will be presented with each imaging modality for each timepoint. The overall protocol timeline is detailed in Figure 1.

#### Neurological Examination and Clinical Rating Scales

We will record the following patient demographics: age, sex, occupation, level of education, ethnicity, languages spoken, handedness, and pre-morbid medical history. We will document the mechanism of injury, severity illness score [SOFA and MODS (26, 27)] injury severity scale (for trauma patients), CT scan and/or MRI findings, clinical EEG/evoked potential findings, the magnitude of intracranial pressure (if monitored, and presence and duration of increased intracranial pressure), sedation/analgesia drug dose equivalents, delirium duration, ICU length of stay, and hospital length of stay. At each imaging time point, we will capture bedside clinical examinations of consciousness using the Glasgow Coma Scale (28), Full Outline of UnResponsiveness Score (FOUR Score) (29), and where feasible, the Coma Recovery Scale-Revised (CRS-R) (30). This will be done immediately before or after imaging occurs.

#### Tracking Neurocognitive and Functional Outcomes

Long-term assessment of neurocognitive function will be obtained through tests of executive function and memory, delivered online via the Cambridge Brain Sciences (CBS) platform (www.cambridgebrainsciences.com) (31, 32). Importantly, the CBS tests have a normative database of over 75,000 participants, as these tests have been taken over 10 million times. The CBS tests will be administered on a laptop or a tablet and are designed to assess deductive and verbal reasoning, episodic memory, visuospatial and working memory, and short-term memory and described in thorough detail elsewhere (33). In brief, these tests are: (1) Spatial Span (short-term memory); (2) Monkey Ladder (visuospatial working memory); (3) Paired Associates; (4) Token Search (working memory and strategy); (5) Odd One Out (deductive reasoning); (6) Rotations (mental rotation); (7) Feature Match (feature-based attention and concentration); (8) Spatial Planning (planning and executive function); (9) Interlocking Polygons (visuospatial processing); (10) Grammatical Reasoning (verbal reasoning); (11) Double Trouble (a modified Stroop task); and (12) Digit Span (verbal working memory) (33).

When patients regain behavioral awareness and are no longer sedated, they will be screened by the bedside nurse daily for delirium, as is the standard of care in our hospital. Participants will be asked to participate in the CBS neurocognitive assessment when they score >4 on the Intensive Care Delirium Screening Checklist (34) indicating that they are not currently experiencing delirium.

Testing will take approximately 20 min to complete. The test series will be given once in the hospital when patients are free of delirium. Following discharge, patients will receive a URL link via email to complete the same test series online monthly for up to 12 months following injury. Standard clinical outcome measures will also be used to track functional outcomes at 3-, 6-, and 12-months post injury through phone interviews using the extended Glasgow Outcome Scale (GOS-E) (35).

#### Data Collection, Management, and Dissemination

Letters of Information and Consent as well as case report forms will be stored in a locked cabinet in the research office. Each patient will have a unique and study-specific identification code that will be assigned to all aspects of testing. The link between the code and the patient's name will be stored in a secure cabinet in the Primary Investigators' research office. Contact and demographic information (name, telephone number, email, date of birth, date of death, sex/gender, age) will be stored on the secure, password-protected server. FMRI, fNIRS, and EEG data will not have any identifiers within the data and stored using the unique code for each healthy control and patient. A unique web link will be sent to patients who choose to complete longitudinal cognitive assessments using CBS via email which will then be linked back to the patient's study ID. All data will be stored at Western University on a secured and encrypted server that is behind institutional firewalls. The results from the neurocognitive test battery will be stored a secure server at the Brain and Mind Institute. We hope that the results from this study will be presented at both local and international scientific research conferences and will be published peer-revived scientific journals.

The results of this study will be not shared with the clinical team and will not inform patient management. In accordance with our local ethical guidelines, we are not permitted to share the results with the clinical team as this study is at the stage of scientific research rather than clinical practice.

### Follow Up Imaging

Patients will be invited to participate in a follow up imaging session approximately 12 months post-injury to assess for changes in brain responses from acute injury to recovery. Imaging paradigms at the follow up scans will be identical to those used during acute injury.

### Stimuli Paradigms

We will first examine neural activity using passive somatosensory and auditory stimuli. These tasks allow for the assessment of both basic and higher order cognitive processing without the need for overt motor action.

#### Somatosensory Perception Paradigm (fMRI/fNIRS)

Somatosensory stimuli will be administered via electrical median nerve stimulation and will be performed simultaneously with neuroimaging testing with the same clinical parameters used in upper limb somatosensory evoked potentials (36). Electrodes will be placed on a patient's arm while in the fMRI suite using a custom-made high-frequency-shielded cable to a battery-powered nerve stimulator (Nikolet VikingQuest EP stimulator). This same cable will also be used to deliver the stimuli at the bedside when recording with fNIRS. The intensity of stimulation will be delivered at a current to induce a continuous motor threshold (approximately 6–30 mA) and kept constant for each patient. The stimuli will consist of 30-s blocks of stimulation at a rate of 4 Hz with a 0.1 ms pulse duration followed by a 30-s interblock rest period. There will be 8 stimulation and 7 rest blocks, with a total test time of 7 min 30 s per hand. We will assess for basic processing in the primary somatosensory cortex and for higher-order activity in the secondary somatosensory cortex and insula. The extent of somatosensory perception (as indexed by neural responses in the primary and secondary somatosensory cortices, and the insula) may be a predictor of neurological outcome.

#### Auditory Processing Paradigm (fMRI/fNIRS/EEG)

In fMRI and fNIRS, a passive hierarchical auditory task will be administered to assess the auditory processing abilities of patients. A subtraction approach (previously described by Coleman and colleagues (11) will be used to measure 3 levels of processing: (i) sensory (ex. speech vs. silence), (ii) perceptual (ex. speech vs. non-intelligible noise, and (iii) semantic (ex. complex narratives vs. pseudowords). Stimuli will be presented in 30-s blocks in an interleaved fashion and each condition will be repeated 5 times for a total duration of 10 min.

In EEG, we will present three different types of auditory stimuli to elicit event related potentials (ERPs) that reflect different levels of auditory function (37). Specifically, we will compare the EEG responses of individual patients collected during silence (rest), signal correlated noise (SCN) created from word stimuli, and word pairs. Word pairs will either be related (e.g., table-*chair*) or unrelated (e.g., dog—*chair*) to elicit ERP components associated with semantic processing. Basic auditory function will be assessed by contrasting grand-average ERP waveforms during silence (rest) and noise, focusing on the N1-P2 complex. Next, we will compare the amplitude and latency of the N1-P2 components between noise and word trials to index automatic speech discrimination. Finally, we will examine differences in ERP amplitude and latency of the N400 response between related and unrelated word pairs. In healthy individuals, related word pairs typically elicit a higher amplitude N400 response which suggests that semantic congruence—a higher-order cognitive process—modulates this ERP component. Stimuli will be presented in 5 min blocks of 50 trials each, with the total duration of testing being under 30 min. Neural activity in response to this hierarchical set of tasks may be a predictor of clinical outcome; that is to say, patients with activation similar to controls may have a more complete recovery from brain injury than those who will show no, or partial, activation.

#### Covert Awareness Paradigm (fMRI/fNIRS/EEG)

While passive paradigms provide insight into the integrity of higher-order cognitive processing in the brain, these stimuli cannot be used to infer conscious awareness as the auditory and somatosensory paradigms can elicit brain activity without any cooperation on the part of the patient. Here, command following paradigms using mental imagery will be used to assess for covert awareness as they produce specific and well documented patterns of neural activity (10, 19). For fMRI and fNIRS, all participants will be given pre–recorded spoken instructions about how to perform two mental imagery tasks (motor imagery and spatial navigation), which have been described in detail previously (10). For the motor imagery task, participants will be instructed to imagine playing a game of tennis, swinging their arm back and forth to hit a ball repeatedly when cued in the scanner by the instruction “imagine playing tennis”. Patients will be instructed to continue to perform the tennis imagery until they hear the words “now just relax.” Prior to the spatial navigation task, individuals will be instructed to imagine moving from room to room in their home and visualize everything they see when cued with the words “imagine moving around your house”. They will be instructed to continue with the task until they hear the instruction “now just relax.” Both paradigms are five and a half minutes in length and have an interleaved block design of 30 s of mental imagery with alternating rest periods (five blocks of imagery, six blocks of rest).

If patients have reliable and appropriate neural activity to the command following tasks, the same motor imagery paradigms will be used as a communication tool with fNIRS at the patient's bedside at a subsequent testing session within 24 h of when covert command following is detected. The patients could be asked to “Imagine playing tennis” if the answer to a question is “yes” and instructed to “imagine walking through your home” if it is “no”. We will begin with asking questions that have correct and objective answers which may include, “does 1lb weight more than 2 lbs?”, “is your last name < insert incorrect last name>?”, “are you in a hospital?”, and “are you in a supermarket?”. Finally, we will ask questions such as “are you in pain?”, “do you feel safe?” to better understand the patients' perceptions of their surroundings and analyze these responses if the objective and verifiable questions are answered correctly.

The lower spatial resolution of EEG requires a slight modification of the mental imagery tasks. In EEG, patients will be asked to imagine squeezing their right hand or moving their toes on both feet each time they hear a beep (19). We will present 15 beeps in a single block and all blocks will end with instructions for the patient to relax. This will be repeated for four to eight blocks total, with hand and toe instructions presented in a random order. This task will last approximately 30 min.

#### Movie Paradigm (fMRI/fNIRS/EEG)

An audio clip from the movie *Taken* will be presented (20, 38). During movie narratives, neural activity synchronizes in sensory regions, coding its perceptual properties, as well as in frontoparietal areas that are associated with the executive processes required to follow a plot. The latter can be used to infer a similar experience (or executive processing) across healthy individuals and can be extended to detect covert cognition in patients who remain behaviorally non-responsive. A “scrambled” version of the audio will also be played that will serve as an auditory control condition. The movie paradigm will be approximately 10 minutes in length. Synchronization of the frontal and parietal cortices to the movie narrative task may be a predictor of neurological outcome.

#### Resting State Paradigm (fMRI/fNIRS/EEG)

A resting-state scan will be acquired to image the brain's intrinsic functional connectivity at rest in the absence of any external stimuli. The resting state scan will be 6 min in length. The extent of functional connections that are preserved in acute brain injury may be a predictor of neurological outcome.

### FMRI Procedures

Participants will be accompanied by a nurse and respiratory technologist during the MRI scanning. The total time of the study will be approximately 60 min. Participants may be removed from the MRI for breaks at any time if necessary. During imaging, patients will be closely monitored by the treating medical team. A total of 6 research scans will be acquired. Imaging data will be acquired on either a 1.5T General Electric MRI machine or a 3T Siemen's scanner located at London Health Sciences Centre (London, Canada). Whole-brain anatomical 3D-SPGR T1-weighted images and functional scan parameters may vary based on the scanner used for imaging.

#### FMRI Data Analysis

fMRI data will be preprocessed and analyzed using Statistical Parametric Mapping (SPM) (http://www.fil.ion.ucl.ac.uk/spm). For the passive paradigms (somatosensory and auditory) as well as the command follow tasks, the analyses will be based on the general linear model (GLM) using the canonical hemodynamic response function. A GLM will be created with regressors for each condition in each active or passive paradigm. Six individual motion parameters will be included as covariates to account for variations due to movement. Healthy control data will be used to generate regions of interest (ROI) masks using a threshold of *p* <0.05, family-wise error corrected. The ROIs will be created using the SPM compatible MarsBaR software (http://marsbar.sourceforge.net/). The statistical threshold to detect neural activity may vary based on the task with supporting evidence from previous literature (10, 11). To assess resting-state functional connectivity, an independent component analysis will be used to decompose the resting state BOLD signal into 20 statistically independent spatial and temporal components with the GIFT software package (http://icatb.sourceforge.net). Components will then be spatially correlated to 10 resting state network templates derived from the BrainMap database (39).

### EEG Procedures

Patients will be fitted with a 128-sensor saline electrolyte electrode cap, from Electrical Geodesics Inc. (EGI). Fitting the cap and checking impedances (below 50 Kohms) will take approximately 15 min. The total time of the testing will be approximately 60 min.

#### EEG Data Analysis

EEG data will be band passed and notch filtered (notch at 60 Hz) and artifacts will be removed using a combination of visual inspection (i.e., of trial epochs), automatic channel rejection, and independent components analysis (ICA). We will examine passive auditory processing using standard ERP analysis techniques (40). These will involve testing the differences in grand average ERP amplitude and latency between silence vs. sound (N1-P2 complex), sound vs. words (modulated N1-P2 or P3), and unassociated word pairs vs. associated word pairs (N400). We will use a correlated components analysis to calculate inter-subject correlations (ISCs) between individual patients and controls during the movie task to assess narrative processing (20). Command following ability will be determined by quantifying systematic decreases in mu (7.5–12.5 Hz) and beta (13–30 Hz) power in scalp regions over the lateral premotor (hand) and medial premotor (toes) cortex (19). For resting state EEG, we will compute inter-electrode magnitude square coherence to examine the functional connectivity profiles of individual patients (41).

### FNIRS Procedures

**Figure 2:**
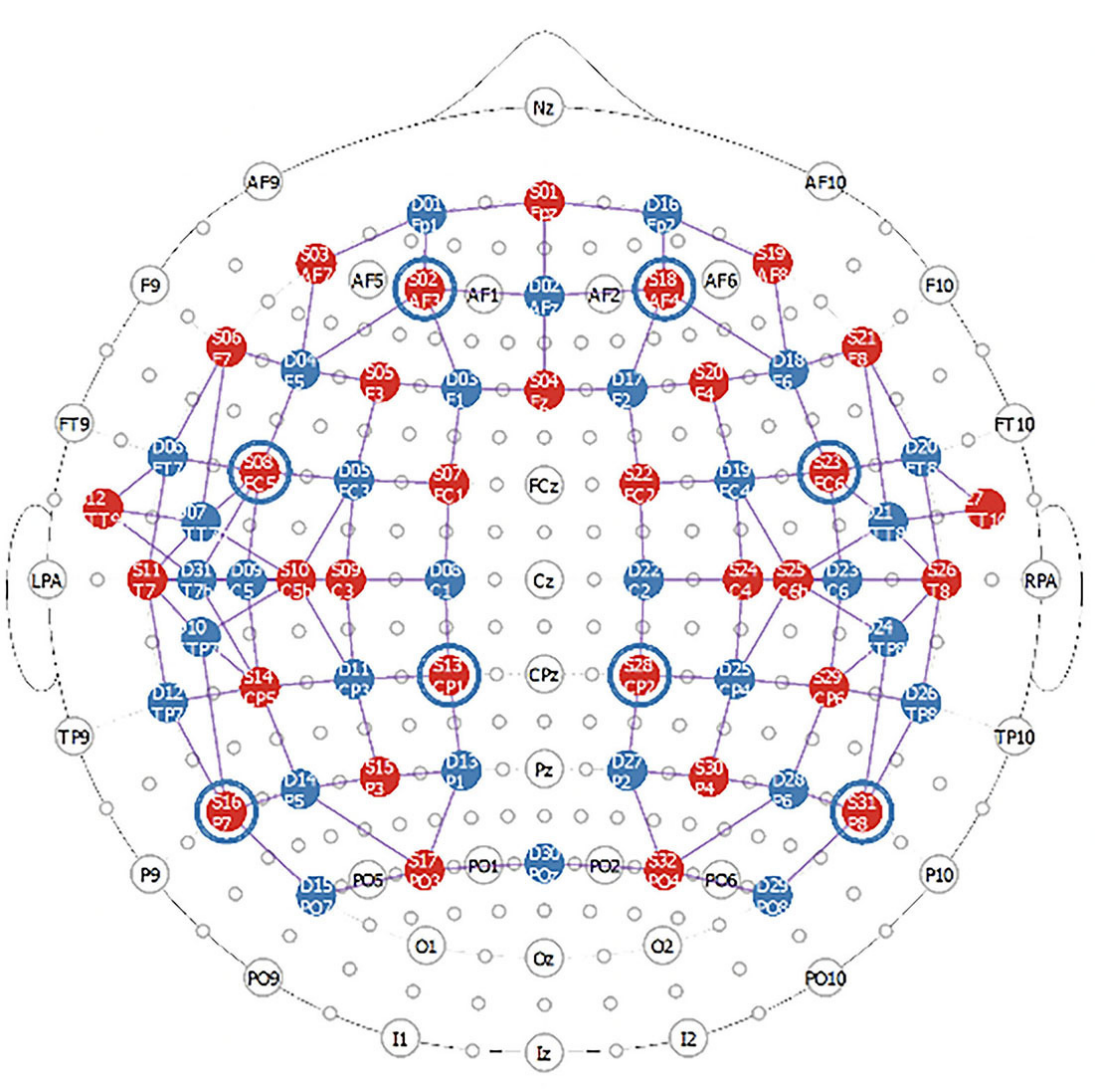
FNIRS montage used in this study. The red circles represent the sources (32 in total) while the blue circles represent the detectors (31 in total). The blue circles around sources 2, 8, 13, 16, 18, 23, 28, 31 represent the location of the short channels which are connected to the last detector (detector 32).

The fNIRS data will be collected with a high-density NIRScout system consisting of 32 sources and 32 detectors (NIRx Medical Technologies). Laser diodes emitting light at four wavelengths (λ = 785, 808, 830 and 850 nm) will be used to measure changes in regional concentrations of oxy- and deoxy-hemoglobin. The sources and detectors will be attached to the head in two bilateral grids with optodes extending from the frontal lobe to the parietal areas. A high-density setup will be used around the primary auditory cortices to enhance the spatial resolution around the superior temporal gyri and around the primary somatosensory cortices in the parietal regions (see Figure 2). Eight short source-detector separations (8 mm) connected to the last detector will be used to monitor scalp effects, while channels with larger source-detector distances (3–4 cm) will measure changes from the brain.

#### FNIRS Data Analysis

The data will be preprocessed and analyzed using MATLAB and freely available software packages such as NIRS Toolbox and Homer2. Each time series will be filtered to eliminate the effects of physiological changes such as heart rate and breathing, corrected for motion artifacts and short-channels will be regressed from the longer channels to reduce scalp contributions (42). For the sensory processing and command following tasks, the time courses will be fitted to the general linear model to detect significant hemoglobin changes. A channel will be considered activated if there is a significant increase in oxyhemoglobin and a concurrent significant decrease in deoxyhemoglobin. Network analysis will be performed for the resting state analysis (43). Pearson correlation coefficient (*r*) between the time-courses of two channels for all possible combinations will be computed, and these will be considered linked if the *r* is above a predetermined threshold. Graph theory will also be used to extract global network parameters to characterize the network.

### Statistical Analysis

To establish whether fMRI can aid prognostication in acute disorders of consciousness, imaging results will be related to long term behavioral and cognitive follow up assessments. For the somatosensory and auditory processing task, the Spearman's rank-order correlation coefficient will be used to measure the rank correlation between the extent of acute sensory processing and a patient's behavioral scores at 3-, 6-, and 12-months post- injury, as indexed by the GOS-E. This same method will be used to evaluate the relationship between the degree of acute functional connectivity, as measured by the correlation value to healthy controls, to long term outcome. For the movie narrative, the correlation between individual patients and the healthy control group during the intact clip will be related to long term outcome. The primary outcome will be 12 months post injury, and the secondary outcomes will be at 3 and 6 months. Linear regressions will be performed to investigate the effect of clinical and demographic variables on acute imaging features and patient outcomes. All statistical tests will be corrected for multiple comparisons using a False Discovery Rate. We will also determine the sensitivity, specificity, positive predictive value, and negative predictive value for the imaging procedures outlined in the protocol.

Receiver operating characteristic (ROC) and area under the curve (AUC) analyses will be generated to identify a cut-off value differentiating conscious from unconscious patients, as well as patients who are to have a good or poor outcome for each paradigm. Outcome predictors (as assessed by the area under a receiver operating characteristic curve) will be compared between acute neuroimaging findings and traditional prognostic markers (such as EEG reactivity, pupillary reflexes, brainstem reflexes, serum biomarkers, evoked potential results) to assess whether functional neuroimaging responses are more accurate. We will account for patient's age, gender, days since injury, down time (for cardiac arrest patients), MODS and SOFA score, as covariates during the statistical analyses. Where possible, patients will be grouped by etiology in the statistical analyses. In line with the previous investigational neuroimaging literature, we will characterize a “good” outcome as a recovery that allows for sufficient function for independent activities of daily life (3, 18, 44), whereas a “poor” outcome will be defined as a severe disability that impacts all aspects of daily living, a coma, vegetative state/unresponsive wakefulness syndrome, and death. In respect to the GOS-E, a “good” outcome is characterized as a score of 4 or greater, signifying the ability to be left alone for up to 8 h during the day without assistance (8).

To establish whether fMRI measures of covert awareness can be used in the diagnosis of acute disorders of consciousness in the ICU, we will seek to ascertain the proportion of patients who show signs of covert awareness, in the form of plot and command following, and subsequently the proportion of those participants who can use mental imagery tasks as a means of communication. A variety of statistical measures will be used, where appropriate, to determine whether neural activity differs between prognostic groups (i.e., survivors vs. non survivors; good outcome vs. poor outcome). Machine learning models will also be applied to evaluate neuroimaging measures as predictors of covert consciousness and subsequent recovery. Features will be extracted from each imaging modality and a two-class linear classifier (such as support vector machines) will be used to classify patients as survivors and non survivors. Patient outcome 12-months post injury will be used as ground truth while training the classifier. Features such as the median change in the signal during the task period and the correlation coefficient between the time series and the theoretical activation model (i.e., box function convolved with a hemodynamic response) will be extracted from the time courses obtained from task-based stimuli. For the resting state task, previous work has shown that the frontoparietal network and the default mode network are critical for consciousness, therefore, the strength of the correlation between channels in these networks will be used to supplement the features extracted from the active tasks. In addition to supervised machine learning, the feasibility of deep learning to predict outcome will also be assessed. The main advantage of deep learning is the ability to extract features that could be overlooked by the user. Given the breadth of data, deep learning approaches such as the convolutional, feedforward, and artificial neural network may be beneficial (45). These options will be further explored once enough data is collected to make this type of analysis feasible. Finally, a feature ranking approach may be used to quantify the importance of each method and metric.

Given that fMRI and fNIRS rely on BOLD imaging principles, we will examine fNIRS results against fMRI findings to assess the accuracy of fNIRS to detect brain activity during various tasks at the bedside. For the tasked based paradigms, ROI will be identified from the fMRI results and the fNIRS channels corresponding to those areas to determine the presence of significant changes in oxy- and deoxyhemoglobin. For the resting state task, the strength of the correlation across functional networks of interest (i.e., frontoparietal network, default mode network) will be compared (15, 17). Furthermore, given that two MRI scanners with different field strengths will be used in this protocol, we will analyze data on the 3T and 1.5T as separate cohorts of patients based on which MRI scanner the data was obtained from. We also plan to address how this will impact the data by running research scans on both scanners in the same group of healthy volunteers. Finally, investigators will be blinded when analyzing predictive data and the study coordinator will be blinded from the acute imaging results when collecting outcome measures.

## Anticipated Results

**Figure 3:**
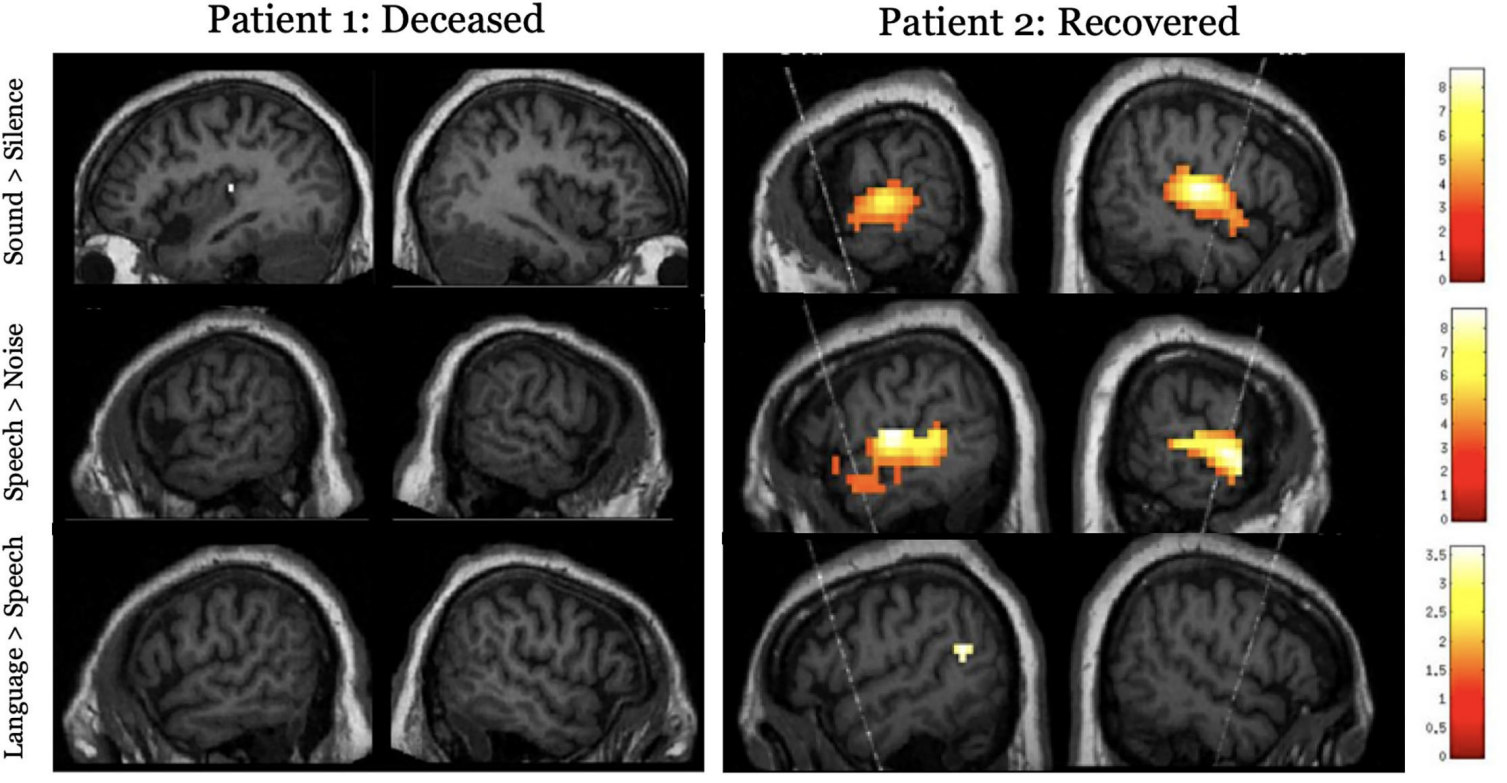
Results for the hierarchical auditory task in two acutely unresponsive patients. The top panel shows activity to the sound perception contrast (sound > silence), the middle panel shows activity to the speech perception contrast (speech > signal correlated noise), and the lower panel assessed language processing (complex language > pseudoword sentences). Results are thresholded at *p* < 0.001, uncorrected and masked inclusively by the healthy control group's results for each respective contrast and displayed on the patient's own structural image.

The overarching hypothesis is that the extent of preserved neural function during task-based paradigms or the degree of functional connectivity/synchronization during rest and the passive movie paradigms will have a positive relationship to GOS-E at 12 months. Specifically, we hypothesize that higher-order auditory and somatosensory processing will be related to better functional outcomes (GOS-E >4 at 12 months post injury), while the absence of low-level sensory processing will be indicative of a poor prognosis. Here, we show two cardiac arrest patients who underwent functional neuroimaging while acutely unresponsive (Figure 3). Patient 1 had no detectable neural responses and did not survive their injury (GOS = 1). Patient 2 exhibited responses to sound and speech that were equivalent to those observed in healthy participants and showed some evidence of language comprehension. This patient regained behavioral awareness and made a good neurological recovery (GOS = 5, 6 months post injury). While no substantive conclusions can be drawn from these results, Figure 3 demonstrates that using neuroimaging tasks are feasible with an acute brain injury patient population.

**Figure 4:**
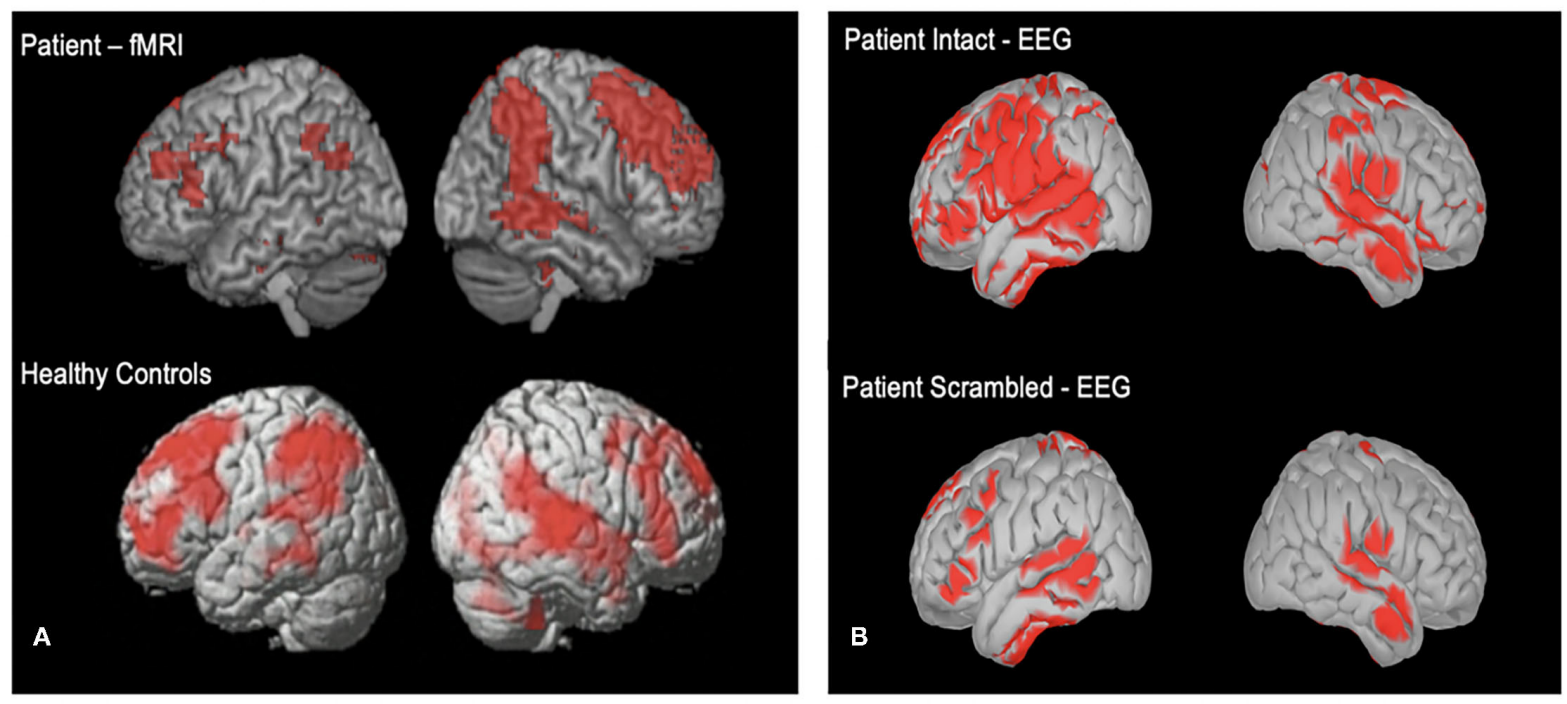
**(A)** One acutely unresponsive patient who underwent fMRI imaging while listening to the soundtrack from part of the movie “Taken”. **(B)** Cortical reconstruction of EEG activity recorded from one unresponsive patient while listening to the “Taken” audio (top) and a scrambled control version of the clip (bottom). EEG activity is source localized following previously described method (17).

We also hypothesize that a small proportion of patients will show evidence of covert awareness by willfully modulating their brain activity in response to command following tasks. Further, we hypothesize that some patients will have synchronized neural activity to the movie narrative task, and the degree of synchronization well relate to long term outcome. Figure 4 shows two comatose patients listening to the soundtrack from part of the movie *Taken*. For both patients, activity in frontal and parietal cortices synchronized significantly with that of healthy controls. Both patients ultimately regained behavioral awareness and were discharged from ICU.

**Figure 5:**
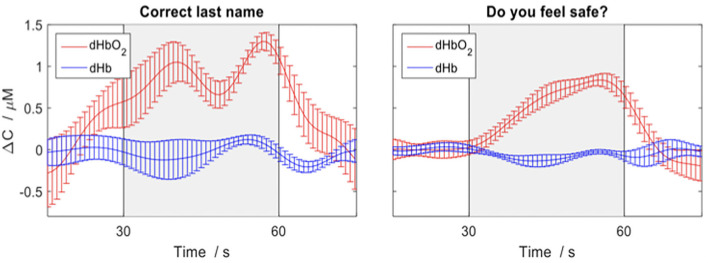
Depicting the changes in concentration of oxygenated (red) and deoxygenated (blue) hemoglobin for various questions average across trials in a behaviorally unresponsive patient in ICU. The response period is indicated by the grey box and the error bars represent the standard error of the mean across channels. An increase in concentration of deoxygenated hemoglobin (red) indicates a yes response. Figure adapted and used with permission (24).

Additionally, we anticipate that fNIRS will allow for communication between brain injured patients who show signs of covert awareness with family and circle of care staff in the ICU. By allowing patients to answer ‘yes' or ‘no' to externally verifiable questions (e.g., own name, current date) using motor imagery tasks, we will be able to communicate with patients about their well-being (e.g., are you in pain?). We have previously demonstrated that fNIRS can be used to communicate in the ICU with a behaviorally unresponsive patient, allowing him to convey information by imagining playing tennis for “yes” and relaxing for “no”, as see in Figure 5 (24).

## Discussion

This research protocol outlines a comprehensive neuroimaging program that aims to improve the diagnosis and prognosis of critically ill patients with a primary brain injury. Functional neuroimaging techniques will be used to (1) identify prognostic markers of good neurologic recovery, (2) develop reliable diagnostic tools for detecting consciousness, and (3) communicate with select patients who show signs of covert awareness. Long term recovery will be tracked using neurocognitive and behavioral tests, and imaging results will be related to these outcome measures to determine which acute features are most predictive of functional outcome.

Ultimately, research derived from this study may be used in establishing new prognostic guidelines for patients with severe brain injury. Current prognostic tools have a primary utility of predicting poor neurologic outcome; however, even the most robust tools provide little insight into the chances of good recovery (46). Neuroimaging methods may be of use in this regard, as they can be used to map changes in the functional and structural integrity of the brain following injury and relate that directly to behavioral outcome. Indeed, a growing number of neuroimaging studies have demonstrated the positive prognostic potential of fMRI, but these results remain to be validated with a larger cohort of patients (17, 47–49). The pressing need for improved prognostic tools is further highlighted by the high variability in the withdrawal of life-sustaining measures across medical centers (50). These differences are guided by physician's perceptions of long-term prognosis (51), further emphasizing the need for accurate and objective prognostic markers. By revealing residual cognitive function in the midst of clinical uncertainty, functional neuroimaging may aid in the prognostic process and therefore affect the outcome of discussions regarding the continuation of care or the withdrawal of life-sustaining measures. The results of this study will not directly inform the prognosis or diagnosis of patients enrolled in study procedures, but rather, may inform future prognostic and diagnostic guidelines for including functional neuroimaging into the standard of care. Importantly, our local ethics research board has restricted the research team from sharing these results with the clinical team as this study is purely scientific and should not directly impact patient care.

We anticipate that this study will have a benefit in assessing the level of consciousness for patients with severe brain injury. Currently, the degree of awareness patients retain while in the ICU is determined solely by their behavioral responses, which are observed at the bedside. However, this method may fail to identify some patients who have absent motor function in response to external stimulation yet may retain covert awareness. Indeed, a recent EEG study demonstrated that 15% of brain injury patients in the ICU who were unable to overtly respond to motor commands had detectable neural activity when given instructions to squeeze their hand (8). Early detection of consciousness is also associated with improved long-term functional outcomes, as time to command following is a powerful predictor of recovery after severe brain (52). Functional neuroimaging methods may complement the current clinical tools used to assess the level of consciousness at the bedside to render a more accurate diagnosis by detecting covert awareness in patients before they show evidence of behavioral responsiveness.

There are many benefits of multi-modal testing in an acute patient setting. The combined use of these modalities (fMRI, EEG, fNIRS) with multiple functional tasks will be more reliable in obtaining a comprehensive insight into the injured brain. Multimodal imaging has been proven to be an effective approach in increasing the likelihood of detecting residual cognitive function in chronic disorders of consciousness (25). Further, repeated testing with fNIRS and EEG at the bedside throughout ICU stay will allow researchers to track how neural activity changes with time. This is especially important when assessing for higher order cognitive function, as levels of awareness can fluctuate during intensive care stay (53). Therefore, repetitive testing is more advantageous than at a single point in time, as the chances of detecting consciousness will be greater over longitudinal testing sessions. To date, only one study has explored whether multimodal techniques like fMRI and EEG combined with a range of paradigms are diagnostically and prognostically useful in the ICU (9). While the results from Edlow and colleagues demonstrate the feasibility of stimulus based multimodal imaging, further systematic study is required to establish the full clinical utility of functional neuroimaging with this patient population.

Determining the true specificity and sensitivity of acute imaging findings will be a notable challenge to this study. Many patients enrolled will likely undergo the withdrawal of life-sustaining measures (WLSM) following a poor prognosis and in keeping with the patient's values where there could be potential for some recovery and thereby confound the determination of these statistical measures. While the proportion of patients who undergo WLSM can vary across the world, a recent Canadian study determined that approximately 70% of deaths in the ICU following brain injury are due to WLSM (50). Due to selection bias, it will not be possible to assess outcomes in patients who undergo WLSM. To address this issue, we will aim to group patients who have died due to withdrawal of care separately from patients who had a natural death when determining which acute imaging markers as most predictive of neurological recovery.

In conclusion, this research program tackles a common, costly, and complex public health problem that currently has no effective solution. Advanced neuroimaging methods will allow researchers and clinicians to understand the neurophysiological processes associated with functional recovery and provide insight into how individuals recover behaviorally while others succumb to injury or remain in a disordered state of consciousness. Ultimately, this study may improve patient care by providing reliable and accurate information regarding neural function, inform future diagnostic and prognostic guidelines as well as improve neurorehabilitation potential by identifying patients who are most likely to recover from acute brain injury.

## Ethics Statement

The studies involving human participants were reviewed and approved by Health Science's Research Ethics Board, Western University. The patients/participants provided their written informed consent to participate in this study.

## Author Contributions

KK and LN wrote the manuscript. AO conceived the study. KK, LN, GL, and AA collected and analyzed the data in this manuscript. SH coordinated the recruitment of patients. All authors contributed to the design of the study and review and editing of the manuscript.

## Funding

This research program was funded and supported by the Canadian Institute of Health Research (CIHR, #408004).

## Conflict of Interest

AO is the Chief Scientific Officer of Cambridge Brain Sciences, of which the cognitive tests used in this study are marketed by. AO and his collaborators are free to use the platform at no cost for their scientific studies. Research projects do not contribute and are not influenced by the company. There exists no overlap between the current study and the activities of Cambridge Brain Sciences, nor is there any cost to the authors, funding bodies, or participants who are involved in the study. The remaining authors declare that the research was conducted in the absence of any commercial or financial relationships that could be construed as a potential conflict of interest.

## Publisher's Note

All claims expressed in this article are solely those of the authors and do not necessarily represent those of their affiliated organizations, or those of the publisher, the editors and the reviewers. Any product that may be evaluated in this article, or claim that may be made by its manufacturer, is not guaranteed or endorsed by the publisher.
